# Early dexamethasone use as a protective measure in non-mechanically ventilated critically ill patients with COVID-19: a multicenter, cohort study

**DOI:** 10.1038/s41598-022-13239-5

**Published:** 2022-06-13

**Authors:** Khalid Al Sulaiman, Ghazwa B. Korayem, Khalid Eljaaly, Ali F. Altebainawi, Omar Al Harbi, Hisham A. Badreldin, Abdullah Al Harthi, Ghada Al Yousif, Ramesh Vishwakarma, Shorouq Albelwi, Rahaf Almutairi, Maha Almousa, Razan Alghamdi, Alaa Alhubaishi, Abdulrahman Alissa, Aisha Alharbi, Rahmah Algarni, Sarah Al Homaid, Khawla Al Qahtani, Nada Akhani, Abdulaleam Al Atassi, Ghassan Al Ghamdi, Ohoud Aljuhani

**Affiliations:** 1 Pharmaceutical Care Department, King Abdulaziz Medical City, Riyadh, Saudi Arabia; 2 College of Pharmacy, King Saud Bin Abdulaziz University for Health Sciences, Riyadh, Saudi Arabia; 3 King Abdullah International Medical Research Center, Riyadh, Saudi Arabia; 4 King Abdulaziz Medical City (KAMC)-Ministry of National Guard Health Affairs (MNGHA), King Abdullah International Medical Research Center/King Saud bin Abdulaziz University for Health Sciences, PO Box 22490, Riyadh, 11426 Saudi Arabia; 5 Saudi Critical Care Pharmacy Research (SCAPE) Platform, Riyadh, Saudi Arabia; 6 Department of Pharmacy Practice, College of pharmacy, Princess Nourah bint Abdulrahman University, P.O. Box 84428, Riyadh, 11671 Saudi Arabia; 7 Department of Pharmacy Practice, Faculty of Pharmacy, King Abdulaziz University, Jeddah, Saudi Arabia; 8 College of Pharmacy, University of Arizona, Tucson, AZ USA; 9 Pharmaceutical Care Services, King Khalid Hospital, Hail Health Cluster, Hail, Saudi Arabia; 10 Statistics Department, European Organization for Research and Treatment of Cancer (EORTC) Headquarters, Brussels, Belgium; 11 Department of Pharmacy Practice, College of Pharmacy, Princess Nourah Bint Abdulrahman University, Riyadh, Saudi Arabia; 12 Pharmaceutical Care Services, King Abdullah bin Abdulaziz University Hospital, Riyadh, Saudi Arabia; 13 Pharmaceutical Care Department, King Abdulaziz University Hospital, Jeddah, Saudi Arabia; 14 Clinical Pharmacy Department, Pharmacy Services Administration, King Fahad Medical City, Riyadh, Saudi Arabia; 15 College of Medicine, King Abdullah International Medical Research Center, King Saud Bin Abdulaziz University for Health Sciences, Riyadh, Saudi Arabia; 16 Intensive Care Department, King Abdulaziz Medical City, Riyadh, Saudi Arabia

**Keywords:** Medical research, Infectious diseases

## Abstract

Dexamethasone showed mortality benefits in patients with COVID-19. However, the optimal timing for dexamethasone initiation to prevent COVID-19 consequences such as respiratory failure requiring mechanical ventilation (MV) is debatable. As a result, the purpose of this study is to assess the impact of early dexamethasone initiation in non-MV critically ill patients with COVID19. This is a multicenter cohort study including adult patients with confirmed COVID-19 admitted to intensive care units (ICUs) and received systemic dexamethasone between March 2020 and March 2021. Patients were categorized into two groups based on the timing for dexamethasone initiation (early vs. late). Patients who were initiated dexamethasone within 24 h of ICU admission were considered in the early group. The primary endpoint was developing respiratory failure that required MV; other outcomes were considered secondary. Propensity score matching (1:1 ratio) was used based on the patient’s SOFA score, MV status, prone status, and early use of tocilizumab within 24 h of ICU admission. Among 208 patients matched using propensity score, one hundred four patients received dexamethasone after 24 h of ICU admission. Among the non-mechanically ventilated patients, late use of dexamethasone was associated with higher odds of developing respiratory failure that required MV (OR [95%CI]: 2.75 [1.12, 6.76], p = 0.02). Additionally, late use was associated with longer hospital length of stay (LOS) (beta coefficient [95%CI]: 0.55 [0.22, 0.88], p = 0.001). The 30-day and in-hospital mortality were higher in the late group; however, they were not statistically significant. In non-mechanically ventilated patients, early dexamethasone use within 24 hours of ICU admission in critically ill patients with COVID-19 could be considered a proactive protective measure.

## Introduction

In 2019, a newly discovered severe acute respiratory syndrome coronavirus 2 (SARS-CoV-2) was first reported in Wuhan, China^1^. Since the outbreak of the virus, more than 509,531,232 confirmed cases have been reported worldwide^2^. More than 6 million deaths occurred among the confirmed SARS-CoV-2 cases with overall case mortality of almost 2% globally^1^. The clinical presentation of patients with COVID-19 ranges in severity from asymptomatic, mild illness to severe pneumonia leading to acute respiratory distress syndrome (ARDS) and associated with a high mortality rate^3^. Severe respiratory symptoms may increase the risk of hospitalization and intensive care unit (ICU) admission^4^. Patients admitted to the ICU with severe acute lung injury had a higher mortality rate, ranging from 26 to 40%^5,6^.

In critically ill patients with severe COVID-19, the hyperactivation of the systematic inflammatory system causes a state known as “cytokine release syndrome” (CRS)^7^. This state may lead to multiple complications such as acute respiratory distress syndrome (ARDS), septic shock, and acute kidney injury (AKI), disseminated intravascular coagulation (DIC), increasing the risk of mortality in those patients^1,8,9^. Currently, several variants of SARS-CoV-2 that cause COVID-19 are discovered. Nevertheless, there are limited treatment options specific for COVID-19. The mainstay for the treatment of patients with moderate to severe COVID-19 is anti-inflammatory/antirheumatic medications, immune-based therapy, antiviral agents, and convalescent plasma^10^.

Given the fact that COVID-19 patients can develop a systemic inflammatory response that can lead to lung injury and multisystem organ dysfunction, corticosteroids (CS) anti-inflammatory effects can serve as a potential therapeutic option^11,12^. The RECOVERY group multicenter, randomized, open-label trial conducted in the United Kingdom showed a significant mortality reduction at 28-days in hospitalized patients who received dexamethasone for up to 10 days compared to patients who received the standard of care^13^. This benefit was observed in mechanically ventilated patients or who required supplemental oxygen at enrollment^13^. Moreover, a systemic review and meta-analysis including 20,197 patients with COVID-19 reported a significant reduction in mortality, ventilator-free days, the number of patients requiring mechanical ventilation for respiratory failure, and the mechanical ventilator duration^14^.

Although most previous studies showed favorable clinical outcomes and mortality benefits in patients who were initiated corticosteroids “early” in the COVID-19 treatment^13,15–17^, the optimal timing for starting corticosteroids in critically ill patients with COVID-19 is still being investigated^18^ especially dexamethasone. Since hyperinflammatory and overreacting immune responses occur early in ARDS presentation, we hypothesized that early initiation of dexamethasone could attenuate the inflammatory process early, leading to survival benefits and reduction in further complications. Therefore, this study evaluates the appropriate timing of systemic dexamethasone initiation in critically ill patients with COVID-19 and its clinical outcomes.

## Methods

### Study design

This was a multicenter, non-interventional cohort study that included critically ill patients aged ≥ 18-years with COVID-19 who received dexamethasone and were admitted to the ICU from March 01, 2020, until March 31, 2021. This study was conducted retrospectively and prospectively. The retrospective component included pre-identified data of COVID-19 positive patients admitted before the date of IRB approval (March–June 2020). While the prospective component was conducted between July 1, 2020 and March 31, 2021.

Patients were diagnosed with COVID-19 using Reverse Transcriptase-Polymerase Chain Reaction (RT-PCR) nasopharyngeal or throat swabs. Eligible patients were then classified into two groups based on the timing of dexamethasone initiation during ICU stay to either “early” or “late.” Patients who were initiated on dexamethasone within 24 h of ICU admission were considered in the early group, while those who received dexamethasone after 24 h of ICU admission were considered a late initiation. All included centers strictly followed the national COVID-19 guidelines for ICU triage, admission, and discharge criteria^19^. The study was approved by King Abdullah International Medical Research Center (KAIMRC) in July 2020 (Ref.# RC20/430/R). King Abdullah International Medical Research Center (KAIMRC)-IRB committee waived the informed consent due to its observational nature. All methods were performed in accordance with relevant guidelines and regulations.

### Settings

The study was conducted at four hospitals in Saudi Arabia: King Abdulaziz Medical City (Riyadh), King Abdulaziz University Hospital (Jeddah), King Abdullah bin Abdulaziz University Hospital (Riyadh), and King Salman Specialist Hospital (Hail). The primary site for this multicenter study was King Abdulaziz Medical City (Riyadh).

### Participants

**Figure 1:**
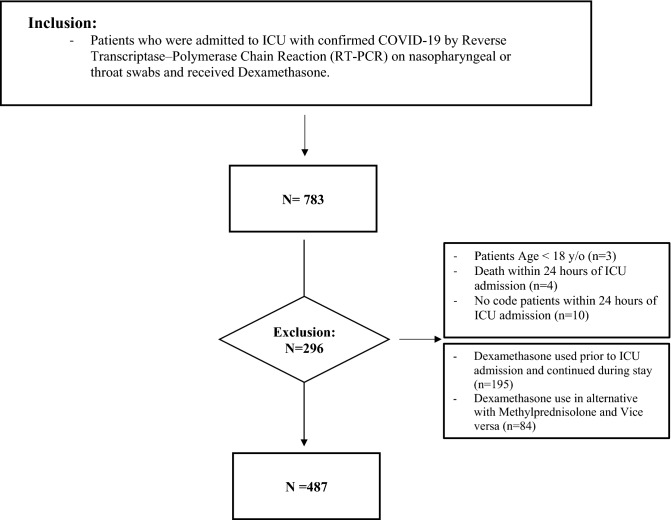
Flow diagram showing patients recruited with COVID-19 who received dexamethasone. *COVID-19* coronavirus disease, *ICU* intensive care unit, *LOS* length of stay.

All included patients have received a dose of 6 mg IV once daily of dexamethasone based on national and international recommendations^13,20^. Exclusion criteria include the initiation of dexamethasone prior to ICU admission, use of dexamethasone interchangeably with methylprednisolone, death within 24 h of ICU admission, or labeled as "Do-Not-Resuscitate" code status within 24 h of ICU admission (Fig. 1).

### Outcomes

The primary endpoint was to assess the association between early dexamethasone initiation and respiratory failure that requires mechanical ventilation in non-MV critically ill patients with COVID-19. The secondary endpoints were 30-day and in-hospital mortality, ICU LOS, hospital LOS, ventilator-free days (VFDs), and complication (s) during ICU stay (i.e., acute kidney injury, liver injury, hospital-acquired pneumonia, secondary fungal infection, and arterial/venous thrombosis).

### Outcome definition(s)

- Respiratory failure was defined as either hypoxemic respiratory failure (PaO_2_ < 60 mmHg with a normal/low arterial carbon dioxide tension (PaCO2)) or hypercapnic respiratory failure (PaCO_2_ > 50 mmHg) that requires mechanical ventilation.
- The 30-day mortality was defined as the in-hospital death occurring for any cause within 30 days of the admission date during a hospital stay; patients who were discharged from the hospital alive before 30 days were presumed to be survivors.
- Ventilator-free days (VFDs) at 30 days were calculated as following: if the patients die within 30 days of MV, the VFDs = 0, VFDs = 30 − days after MV initiation (if patient survived and was successfully liberated from MV), and VFDs = 0 if the patient is on MV for > 30 days.
- Acute kidney injury (AKI) was defined as a sudden decrease of renal function within 48 h, defined by an increase in absolute SCr of at least 26.5 μmol/L (0.3 mg/dL) or by a percentage increase in SCr ≥ 50% (1.5 × baseline value) during ICU stay^21^.
- Acute liver injury was defined as alanine aminotransferase (ALT) exceeding three times the upper limit of normal or double in patients with elevated baseline ALT during the ICU stay.
- Secondary fungal infection was identified through blood, urine, wound, drainage, cerebrospinal fluid (CSF), and/or respiratory cultures. Fungal growth was considered significant if the growth was ≥ 100,000 CFU/ml in sputum or endotracheal aspiration if bronchoalveolar lavage (BAL) shows growth of ≥ 10,000 CFU of single organism/ml for protected specimen brushes (PSBs), and ≥ 100,000 CFU of single organism/ml for BAL fluid. Additionally, urinary cultures were considered significant if showing a growth of ≥ 100,000 CFU/ml of no more than two species of microorganisms. Cultures were excluded if the laboratory reported them as a "contaminant sample”^22,23^.
- Arterial/venous thrombosis was defined using the International Classification of Diseases, Tenth Revision, Clinical Modification (ICD10-CM) code (i.e., myocardial infarction (MI), ischemic stroke, pulmonary embolism, deep vein thrombosis)^24^.

### Data collection

Study data were collected and managed using Research Electronic Data Capture (REDCap^®^) software hosted by King Abdullah International Medical Research Center (KAIMRC)^25,26^. Data collected include patients' demographic information (see additional file 1), comorbidities, vital signs, and laboratory data. In addition, severity scores (i.e., Acute Physiology and Chronic Health Evaluation II (APACHE II), Sequential Organ Failure Assessment (SOFA)), Glasgow Coma Score (GCS), acute kidney injury, proning position status, the needs for mechanical ventilation (MV) and MV parameters (e.g., lowest PaO_2_/FiO_2_ ratio, highest FiO_2_ requirement) within 24 h of ICU admission. Also, renal profile (e.g. serum creatinine, blood urea nitrogen (BUN)), liver function tests (LFTs), coagulation profile (i.e., INR, aPTT, fibrinogen), and inflammatory markers (e.g. C-reactive protein (CRP)) within 24 h of ICU admission were documented. Tocilizumab use was recorded for the eligible patients. All patients were followed until they were discharged from the hospital or died during their hospital stay.

### Statistical analysis

Categorical variables were presented as number (percentage), continuous variables as mean and standard deviation (SD), or median with lower and upper quartile (Q1, Q3) as appropriate. The normality assumptions were assessed for all numerical variables using a statistical test (i.e., Shapiro–Wilk test) and graphical representation (i.e., histograms and Q-Q plots). We assessed model fit using the Hosmer–Lemeshow goodness-of-fit test.

Categorical variables were compared using Chi-square or Fisher exact test. We compared normally distributed continuous variables using student t-test and other non-normally distributed continuous variables with Mann–Whitney U test. Baseline characteristics, baseline severity, and outcome variables were compared between the two groups. Model fit was assessed using the Hosmer–Lemeshow goodness-of-fit test. We aimed to enroll as many patients as possible with no predefined sample size. For the primary outcome, non-mechanically ventilated patients were used as the denominator. Multivariable logistic and negative binomial regression analysis were used for the outcomes considered in this study and reported using the odds ratios (OR) or estimates with the 95% confidence intervals (CI) as appropriate. Regression analysis was done by considering the PS score as one of the covariates in the model. Additionally, Kaplan–Meier (KM) plots were generated for the mortality during hospital stay. No imputation was made for missing data as the cohort of patients in our study was not derived from random selection. We considered a* P* value of < 0.05 statistically significant and used SAS version 9.4 for all statistical analyses.

Propensity score matching procedure (Proc PS match) (SAS, Cary, NC) was used to match patients who received late dexamethasone (active group) to patients who received early dexamethasone (control group) based on patient’s SOFA score, mechanical ventilation (MV) status, proning status, and early use of tocilizumab within 24 hours of ICU admission (1:1 ratio). A greedy nearest neighbor matching method was used in which one patient who received late dexamethasone (active group) matched with one patient who received early dexamethasone (control group), which eventually produced the smallest within-pair difference among all available pairs with treated patients. Patients were matched only if the difference in the logits of the propensity scores for pairs of patients from the two groups was less than or equal to 0.5 times the pooled estimate of the standard deviation.

### Ethics approval and consent to participate

The study was approved in July 2020 by King Abdullah International Medical Research Center Institutional Review Board, Riyadh, Saudi Arabia (Reference No: RC20/430/R). King Abdullah International Medical Research Center (KAIMRC)-IRB committee waived the informed consent due to the research's method as per the policy of the governmental and local research center. Participants’ confidentiality was strictly observed throughout the study by using anonymous unique serial number for each subject and restricting data only to the investigators. All methods were performed in accordance with relevant guidelines and regulations.

## Results

### Demographic and clinical characteristics

A total of 783 critically ill patients with COVID-19 who received dexamethasone were screened; 487 patients were included during the study period. Out of the 487 patients, 76.4% (372 patients) received dexamethasone early within 24 h of ICU admission. A total of 208 patients were included after propensity score matching (1:1 ratio) based on the selected criteria (Fig. 1). The majority of the patients were men (70.1%), and the mean age of the patients was 62.3 ± 14.8 years. The most common comorbidities before PS matching were diabetes mellitus (DM) (61.5%), hypertension (HTN) (56.9%), and dyslipidemia (DLP) (17.7%); comorbidities were not significantly different between the two groups except for stroke, as presented in Table E1 available in the online supplement.

There was no significant difference between the two groups’ baseline severity scores (i.e., APACHE II, and SOFA scores), the needs for MV within 24 h (early: 61.5% vs. late: 59.6%, p = 0.77), baseline Oxygenation Index (OI) (early: 16.3 vs. late: 17.3, p = 0.30), PaO_2_/FiO_2_ ratio (median 83.1 in the early group vs. 79.5 in the late group, p = 0.55), blood glucose level, lactic acid, platelets count, CRP, Creatine phosphokinase (CPK), ferritin, procalcitonin, eGFR and AKI status within 24 h of ICU admission (early 31.7% vs. late 26.9%, p = 0.4463) after using propensity score. The median APACHE II score was 13, while the median SOFA score was 4 in patients who received dexamethasone after 24 h of ICU admission. Additionally, the early use of tocilizumab within 24 h of ICU admission was similar between the two groups (6.7%; p = 0.99) (Table E1).

### Respiratory failure and mortality

Among the non-MV critically ill patients with COVID-19, late initiation of dexamethasone was associated with higher odds of developing respiratory failure that required MV support (OR (95%CI): 2.75 (1.12,6.76), p = 0.02) as demonstrated in Table 1.

**Table 1:** Regression analysis of complications during ICU stay after propensity score.

Outcomes	n of outcomes/total no-of patients		P-value	Odds ratio (OR) (95%CI)	P-value^d^
	Early	Late			
Respiratory failure required MV, n (%)^a^	16/40 (40.0)	27/42 (64.3)	0.03^c^	2.75 (1.12, 6.76)	0.02
Acute kidney injury, n(%)^b^	48 (46.2)	43 (41.3)	0.48^c^	0.82 (0.47, 1.43)	0.49
Liver injury, n(%)^b^	11 (10.6)	8 (7.7)	0.47^c^	0.70 (0.27, 1.83)	0.47
Thrombosis/infarction, n(%)^b^	6 (5.8)	7 (6.7)	0.77^c^	1.02 (0.39, 2.67)	0.98
Hospital acquired pneumonia, n(%)^b^	7 (6.7)	10 (9.6)	0.45^c^	1.49 (0.54, 4.12)	0.44
Secondary fungal infection, n(%)^b^	7 (9.2)	9 (10.5)	0.79^c^	1.15 (0.41, 3.25)	0.79

^a^Denominator of the percentage is non-mechanically ventilated patients with 24 h of ICU admission.

^b^Denominator of the percentage is the total number of patients.

^c^Chi-square test is used to calculate the P-value.

^d^Propensity score matched used based on patient’s SOFA score, MV within 24 h of ICU admission, proning position, and early use of Tocilizumab within 24 h of ICU admission.

**Figure 2:**
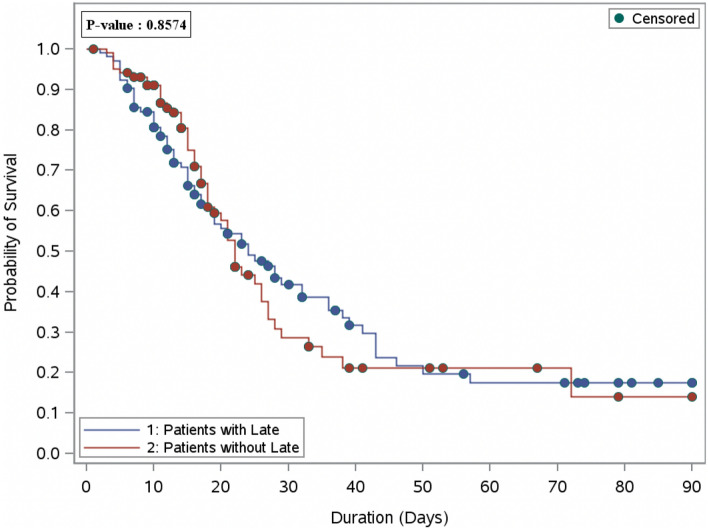
Overall survival plot during the hospital stay after PS matching comparing patients who received late dexamethasone (104 patients) versus the early dexamethasone (104 patients).

In crude analysis, the 30-day mortality was 50.5% in the early group compared to 57.7% in the late initiation (p-value = 0.29). Moreover, the 30-day mortality was not statistically significant between the two groups in the regression analysis the (OR [95%CI]: 1.34 [0.77, 2.32], p = 0.29). Furthermore, the in-hospital mortality was 51.5% in the early group versus 62.5% in the late group, which was 1.6 folds higher at logistic regression analysis in patients who received dexamethasone after 24 h of ICU admission (OR [95 percent CI]: 1.57 [0.90, 2.74], p = 0.11); however, it was not statistically significant (Table 2). Additionally, the overall survival probabilities were similar between the two groups during hospital stay after the propensity score matching, as presented in the survival curve (Fig. 2).

**Table 2:** Regression analysis of mortality, VFDs, and length of stay after propensity score.

Outcomes	Early	Late		Odds ratio (OR) (95%CI)	P-value^f^
			P-value		
30-day mortality, n (%)^a^	52 (50.5)	60 (57.7)	0.29^d^	1.34 (0.77, 2.32)	0.29
In-hospital mortality, n (%)^a^	53 (51.5)	65 (62.5)	0.11^d^	1.57 (0.90, 2.74)	0.11

				Beta coefficient (estimates) (95%CI)	P-value^f^
Ventilator free days (VFDs), mean (SD)^a^	10.8 (13.26)	7.7 (11.19)	0.08^c^	− 0.31 (− 1.05, 0.43)	0.42
ICU length of stay (days), median (Q1,Q3)^b^	7.5 (4.00, 13.00)	12.0 (8.00, 20.00)	0.01^c^	0.28 (− 0.06, 0.62)	0.11
Hospital length of stay (days), median (Q1,Q3)^b^	15.0 (10.00, 22.00)	21.0 (12.00, 39.00)	0.04^c^	0.55 (0.22, 0.88)	0.001

^a^Denominator of the percentage is the total number of patients.

^b^Denominator is patients who survived.

^c^Wilcoxon rank sum test is used to calculate the P-value.

^d^Chi-square test is used to calculate the P-value.

^f^Propensity score matched used based on patient’s SOFA score, MV within 24 h of ICU admission, proning position, and early use of Tocilizumab within 24 h of ICU admission.

### Complications and the length of stay

Complication during ICU stay such as hospital acquired pneumonia (OR (95%CI): 1.49 (0.54, 4.12), p = 0.44), secondary fungal infection (OR (95%CI): 1.15 (0.41, 3.25), p = 0.79), acute kidney injury (AKI) (OR (95%CI): 0.82 (047, 1.43), p = 0.49), liver injury (OR (95%CI): 0.70 (0.27, 1.83), p = 0.47) and thrombosis (OR (95%CI): 1.02 (0.39, 2.67), p = 0.98) were not statistically significant between the two groups as shown in Table 1.

Among surviving patients who received late dexamethasone, we observed a significantly longer hospital LOS compared to the early initiation (beta coefficient [95%CI]: 0.55 [0.22, 0.88], p = 0.001). Moreover, late initiation of dexamethasone was associated with significantly shorter VFDs (beta coefficient [95%CI]: − 0.31 [− 1.05, 0.43], p = 0.42); however, it did not reach statistical significance (Table 2).

### Follow-up inflammatory/surrogate markers during the stay

Most of the follow-up inflammatory/surrogate markers during the stay (i.e., D-dimer and CPK) were the same between the two groups, except that ferritin peak level was significantly higher in patients who received dexamethasone after 24 h of ICU admission in comparison to the early initiation with a beta coefficient (95%CI): 0.33 (0.01, 0.65), p = 0.04 (Table 3).

**Table 3:** Regression analysis of peak inflammatory/surrogate markers after propensity score.

Inflammatory/surrogate markers	Early	Late	P-value^a^	Beta coefficient (estimates) (95%CI)	P-value^b^
Ferritin level (peak), mean (SD))	1616.5 (1333.75)	2250.7 (4883.26)	0.37	0.33 (0.01, 0.65)	0.04
D-dimer level (peak), mean (SD)	4.7 (13.30)	3.5 (6.33)	0.64	− 0.22 (− 0.83, 0.39)	0.48
Creatine phosphokinase (CPK) level (peak), mean (SD)	1132.8 (4711.68)	924.4 (2188.64)	0.76	− 0.29 (− 0.73, 0.14)	0.19

^a^Wilcoxon rank sum test is used to calculate the P-value.

^b^Propensity score matched used based on patient’s SOFA score, MV within 24 h of ICU admission, proning position, and early use of Tocilizumab within 24 h of ICU admission.

## Discussion

Our study aimed to assess the time of dexamethasone initiation on the clinical outcomes of critically ill patients with COVID-19. Dexamethasone was initiated early in most of the included patients (76.4%). This study was conducted before releasing RECOVERY Collaborative group study results about dexamethasone use in COVID-19^13^. Due to inconsistent evidence about steroids use in COVID-19 at that time, some clinicians were hesitant to start early dexamethasone in all patients. After propensity score matching using the SOFA score, MV status within 24 hours of ICU admission, proning position status, and tocilizumab use within 24 hours of ICU admission, we found that dexamethasone early initiation was associated with lower respiratory failure that required MV support. Additionally, early initiation was associated with shorter hospital LOS among the survived patients.

In critically ill patients with COVID-19, the dysregulated inflammatory immune response observed can be counteracted by using corticosteroids to down-regulate the inflammatory immune response and accelerate disease resolution^27,28^. Although the World Health Organization (WHO) initially did not recommend using corticosteroids for COVID-19 treatment, as of September 02, 2020, the WHO and the National Institute of Health (NIH) recommended using systemic corticosteroids in critically ill patients with severe COVID-19^29,30^. Moreover, the use of dexamethasone is recommended by the surviving sepsis guideline for patients with severe COVID-19 requiring MV and patients with refractory shock^31^. However, none of these guidelines recommend the appropriate time for corticosteroid initiation in critically ill patients with COVID-19.

In our study, early dexamethasone initiation was associated with a significant reduction in respiratory failure that required MV support among non-MV patients. Besides the mortality benefits observed with dexamethasone use, limited studies investigated the impact of late corticosteroid initiation on respiratory failure that required MV. Even though the NIH guideline recommends using corticosteroids in severely ill COVID-19 patients who require MV, our study found that early initiation of dexamethasone was associated with a lower odd of respiratory failure that required MV in non-MV patients^13,29^. That could be related to dexamethasone's prolonged and potent effect, which can mediate systemic and pulmonary inflammation downregulation, restore homeostasis, and enhance disease resolution. In parallel to our findings, Monedero et al. reported a lower MV rate in the early steroids group^16^. The cutoff for early corticosteroid initiation in that study was 48 hours after ICU admission, which included patients who were initiated on corticosteroids prior to ICU admission as well as patients who received other corticosteroid types such as methylprednisolone.

The ICU mortality rate in our study, particularly by day 90, was remarkably high. This finding might partially be explained by the timing of data collection earlier in the pandemic when many patients were not adequately vaccinated. Our results showed a higher 30-day and in-hospital mortality in the late initiation group yet were not statistically significant. Previous studies have found mortality benefits when comparing corticosteroid use to the standard of care alone^13,14,32^. In the RECOVERY trial, dexamethasone compared to standard of care was associated with lower 28-day mortality among hospitalized patients with COVID-19 who received invasive mechanical ventilation or oxygen but not those who did not need respiratory support^13^. Suggesting that the benefit of dexamethasone in patients with COVID-19 is noticeable receiving dexamethasone more than seven days post-symptoms onset^13^.

Additionally, two meta-analyses and systemic reviews reported a significant reduction in the mortality rates in patients with COVID-19 receiving corticosteroids compared to standard care only^14,32^. However, none of these studies compared patients who received early dexamethasone to those who received it late in terms of respiratory failure required MV, mortality benefits, length of stay (LOS), or MV duration. On the other hand, an observational retrospective study including 615 patients with COVID-19 found that starting corticosteroids at > 72 h from admission was associated with significant mortality reduction (HR 0.56, 95% CI 0.38–0.82; p = 0.003) compared to earlier initiation of corticosteroids (within 24 h)^33^. It is noteworthy that most of the study patients received methylprednisolone (87%) instead of dexamethasone^33^.

We believe that our multicenter non-interventional cohort study is one of the first studies to highlight the appropriate time of dexamethasone initiation and its effects on the clinical outcomes of critically ill patients with COVID-19. Its prospective component allows us to prospectively explore the association between the time of dexamethasone therapy initiation in COVID-19 patients with ICU mortality. It also included a predetermined cutoff range for early vs. late initiation time, and the final analysis assessed a variety of important clinical outcomes. Nevertheless, we also determined some limitations to our study. First, the observational nature of the study design limits the exclusion of missing data of some variables. Second, some residual confounding factors are still possible despite propensity score matching. Lastly, there was a dynamic change in the national and international COVID-19 management guidelines as more evidence emerged, affecting the general practice.

## Conclusion

Early use of dexamethasone within 24 hours of ICU admission in critically ill patients with COVID-19 could be considered a proactive protective measure in non-MV critically ill patients with COVID-19. Thus, might minimize the need for mechanical ventilation support. Further randomized clinical and interventional studies are needed to confirm the optimal timing for corticosteroid initiation and its benefit in non-MV critically ill patients with COVID-19.

## Supplementary Information

Supplementary Table 1.

## Data Availability

The datasets used and/or analyzed during the current study are available from corresponding author on reasonable request.

## References

[1] Guan W, Ni Z, Hu Y, Liang W, Ou C, He J, Liu L, Shan H, Lei C, Hui DSC, Du B, Li L, Zeng G, Yuen K-Y, Chen R, Tang C, Wang T, Chen P, Xiang J, Li S, Wang J, Liang Z, Peng Y, Wei L, Liu Y, Hu Y, Peng P, Wang J, Liu J (2020). Clinical characteristics of coronavirus disease 2019 in China. N. Engl. J. Med..

[2] World Health Organization. WHO Coronavirus (COVID-19) Dashboard. https://covid19.who.int/.

[3] Wu Z, McGoogan JM (2020). Characteristics of and important lessons from the coronavirus disease 2019 (COVID-19) outbreak in China. JAMA.

[4] Grasselli G, Zangrillo A, Zanella A, Antonelli M, Cabrini L, Castelli A, Cereda D, Coluccello A, Foti G, Fumagalli R, Iotti G, Latronico N, Lorini L, Merler S, Natalini G, Piatti A, Ranieri MV, Scandroglio AM, Storti E, Cecconi M, Pesenti A, Agosteo E, Alaimo V, Albano G, Albertin A, Alborghetti A, Aldegheri G, Antonini B, Barbara E (2020). Baseline characteristics and outcomes of 1591 patients infected with SARS-CoV-2 admitted to ICUs of the Lombardy region, Italy. JAMA.

[5] Zhou F, Yu T, Du R, Fan G, Liu Y, Liu Z, Xiang J, Wang Y, Song B, Gu X, Guan L, Wei Y, Li H, Wu X, Xu J, Tu S, Zhang Y, Chen H, Cao B (2020). Clinical course and risk factors for mortality of adult inpatients with COVID-19 in Wuhan, China: A retrospective cohort study. Lancet.

[6] Al Sulaiman KA, Aljuhani O, Eljaaly K, Alharbi AA, Al Shabasy AM, Alsaeedi AS, Al Mutairi M, Badreldin HA, AlHarbi SA, Al Haji HA, Al Zumai OI, Vishwakarma RK, Alkatheri A (2021). Clinical features and outcomes of critically ill patients with coronavirus disease 2019 (COVID-19): A multicenter cohort study. Int. J. Infect. Dis..

[7] Coomes EA, Haghbayan H (2020). Interleukin-6 in COVID-19: A systematic review and meta-analysis. medRxiv.

[8] Yang X, Jin Y, Li R, Zhang Z, Sun R, Chen D (2020). Prevalence and impact of acute renal impairment on COVID-19: A systematic review and meta-analysis. Crit. Care.

[9] Ahmad I, Rathore FA (2020). Neurological manifestations and complications of COVID-19: A literature review. J. Clin. Neurosci..

[10] National Institutes of Health. NIH COVID-19 Treatment Guidelines: Pharmacologic Interventions. https://www.covid19treatmentguidelines.nih.gov/management/critical-care/pharmacologic-interventions/.

[11] Arabi YM, Chrousos GP, Meduri GU (2020). The ten reasons why corticosteroid therapy reduces mortality in severe COVID-19. Intensive Care Med..

[12] Pujari R, Thommana MV, Ruiz Mercedes B, Serwat A (2020). Therapeutic options for COVID-19: A review. Cureus.

[13] RECOVERY Collaborative Group (2021). Dexamethasone in hospitalized patients with covid-19. N. Engl. J. Med..

[14] van Paassen J, Vos JS, Hoekstra EM, Neumann KMI, Boot PC, Arbous SM (2020). Corticosteroid use in COVID-19 patients: A systematic review and meta-analysis on clinical outcomes. Crit. Care.

[15] Hyun JH, Kim MH, Sohn Y, Cho Y, Baek YJ, Kim JH, Ahn JY, Choi JY, Yeom JS, Ahn MY, Kim EJ, Baek J-H, Kim YK, Choi H, Jeong SJ (2021). Effects of early corticosteroid use in patients with severe coronavirus disease 2019. BMC Infect. Dis..

[16] Monedero P, Gea A, Castro P, Candela-Toha AM, Hernández-Sanz ML, Arruti E, Villar J, Ferrando C (2021). Early corticosteroids are associated with lower mortality in critically ill patients with COVID-19: A cohort study. Crit. Care.

[17] Fadel R, Morrison AR, Vahia A, Smith ZR, Chaudhry Z, Bhargava P, Miller J, Kenney RM, Alangaden G, Ramesh MS, Nauriyal V, Lakshmikanth J, Abdul Hamed A, Nadeem O, Griebe K, Johnson JM, Bradley P, Uduman J, Hegab S, Swiderek J, Godfrey A, Jennings J, Gardner-Gray J, Ackerman A, Lezotte J, Ruhala J, Samuel L, Tibbetts RJ, Brar I (2020). Early short-course corticosteroids in hospitalized patients with COVID-19. Clin. Infect. Dis..

[18] Timing of Corticosteroids in COVID-19. https://clinicaltrials.gov/ct2/show/NCT04530409.

[19] *ICU triage, admission, and discharge criteria during the Covid 19 pandemic* . Ministry of Health (MOH). (n.d.). (Accessed 4 May 2022) https://www.moh.gov.sa/Ministry/MediaCenter/Publications/Documents/ICU-Criteria-during.pdf.

[20] Saudi Ministry of Health. *Saudi MoH Protocol for Patients Suspected of/Confirmed with COVID-19: Supportive care and antiviral treatment of suspected or confirmed COVID-19 infection (version 2.0) June 17th*. (2020). https://covid19.cdc.gov.sa.

[21] Lin C-Y (2012). Acute kidney injury classification: AKIN and RIFLE criteria in critical patients. World J. Crit. Care Med..

[22] Aleidan FAS, Alkhelaifi H, Alsenaid A, Alromaizan H, Alsalham F, Almutairi A, Alsulaiman K, Abdel Gadir AG (2021). Incidence and risk factors of carbapenem-resistant *Enterobacteriaceae* infection in intensive care units: A matched case–control study. Expert Rev. Anti Infect. Ther..

[23] Koenig SM, Truwit JD (2006). Ventilator-associated pneumonia: Diagnosis, treatment, and prevention. Clin. Microbiol. Rev..

[24] ICD-ICD-10-CM-International Classification of Diseases, Tenth Revision, Clinical Modification. (2021).

[25] Harris PA, Taylor R, Thielke R, Payne J, Gonzalez N, Conde JG (2009). Research electronic data capture (REDCap)—A metadata-driven methodology and workflow process for providing translational research informatics support. J. Biomed. Inform..

[26] Harris PA, Taylor R, Minor BL, Elliott V, Fernandez M, O’Neal L, McLeod L, Delacqua G, Delacqua F, Kirby J, Duda SN, REDCap Consortium (2019). The REDCap consortium: Building an international community of software partners. J. Biomed. Inform..

[27] Mehta P, McAuley DF, Brown M, Sanchez E, Tattersall RS, Manson JJ (2020). COVID-19: Consider cytokine storm syndromes and immunosuppression. Lancet.

[28] Jamilloux Y, Henry T, Belot A, Viel S, Fauter M, el Jammal T, Walzer T, François B, Sève P (2020). Should we stimulate or suppress immune responses in COVID-19? Cytokine and anti-cytokine interventions. Autoimmun. Rev..

[29] National Isntitutes of Health. NIH Coronavirus Disease 2019 (COVID-19) Treatment Guidelines. https://www.covid19treatmentguidelines.nih.gov/. 34003615

[30] COVID-19 Clinical management: Living Guidance, 25 January 2021. https://www.who.int/publications/i/item/WHO-2019-nCoV-clinical-2021-1.

[31] Alhazzani W, Møller MH, Arabi YM, Loeb M, Gong MN, Fan E, Oczkowski S, Levy MM, Derde L, Dzierba A, Du B, Aboodi M, Wunsch H, Cecconi M, Koh Y, Chertow DS, Maitland K, Alshamsi F, Belley-Cote E, Greco M, Laundy M, Morgan JS, Kesecioglu J, McGeer A, Mermel L, Mammen MJ, Alexander PE, Arrington A, Centofanti JE (2020). Surviving sepsis campaign: Guidelines on the management of critically ill adults with Coronavirus Disease 2019 (COVID-19). Intensive Care Med..

[32] Sterne JAC, Murthy S, Diaz JV, Slutsky AS, Villar J, Angus DC, Annane D, Azevedo LCP, Berwanger O, Cavalcanti AB, Dequin P-F, Du B, Emberson J, Fisher D, Giraudeau B, Gordon AC, Granholm A, Green C, Haynes R, Heming N, Higgins JPT, Horby P, Jüni P, Landray MJ, Le Gouge A, Leclerc M, Lim WS, Machado FR, McArthur C (2020). Association between administration of systemic corticosteroids and mortality among critically ill patients with COVID-19. JAMA.

[33] Bahl A, Johnson S, Chen N-W (2021). Timing of corticosteroids impacts mortality in hospitalized COVID-19 patients. Intern. Emerg. Med..

