# Supplementary material for: Early dexamethasone use as a protective measure in non-mechanically ventilated critically ill patients with COVID-19: a multicenter, cohort study
Source: Sci Rep. 2022 Jun 13;12:9766. doi: 10.1038/s41598-022-13239-5 (PMC9191551; doi:10.1038/s41598-022-13239-5)
Supplement: Supplementary file 1 — Supplementary Table 1. [file 41598_2022_13239_MOESM1_ESM.docx]

**Additional file 1: Table E1 - Summary of Demography and Baseline characteristics**

|  | **Before propensity score (PS) adjustment** | | | | **After propensity score (PS) adjustment** | | | |
| --- | --- | --- | --- | --- | --- | --- | --- | --- |
|  | **Overall (N=487)** | **Early (N=372)** | **Late (N=115)** | **P-value** | **Overall (N=208)** | **Early (N=104)** | **Late (N=104)** | **P-value** |
| **Age (Years), Mean (SD)** | 62.3 (14.78) | 61.9 (14.98) | 63.8 (14.10) | 0.2286^ | 62.9 (15.33) | 62.3 (16.44) | 63.5 (14.19) | 0.5762* |
| **Gender – Male, n (%)** | 333 ( 70.1 ) | 252 ( 69.6 ) | 81 ( 71.7 ) | 0.6750^^ | 152 ( 73.1 ) | 77 ( 74.0 ) | 75 ( 72.1 ) | 0.7546^^ |
| **Weight (kg), Mean (SD)** | 81.4 (19.59) | 81.3 (19.16) | 81.7 (21.02) | 0.8549^ | 80.5 (18.39) | 78.6 (14.90) | 82.5 (21.22) | 0.3731^ |
| **APACHE II score, Median (Q1,Q3)** | 13.0 (9.00, 22.00) | 13.0 (9.00, 23.00) | 13.0 (8.00, 20.00) | 0.2505^ | 13.0 (8.00, 20.00) | 13.0 (9.00, 21.00) | 13.0 (8.00, 20.00) | 0.7216^ |
| **SOFA score, Median (Q1,Q3)** | 5.0 (3.00, 7.00) | 5.0 (3.00, 8.00) | 4.0 (3.00, 7.00) | 0.1007^ | 4.0 (3.00, 7.00) | 5.0 (3.00, 7.00) | 4.0 (3.00, 7.00) | 0.7423^ |
| **Early use of Tocilizumab within 24 hours of admission, n (%)** | 73 ( 15.4 ) | 64 ( 17.7 ) | 9 ( 8.0 ) | 0.0135^^ | 14 ( 6.7 ) | 7 ( 6.7 ) | 7 ( 6.7 ) | >0.9999^^ |
| **Prone status, n (%)** | 149 ( 31.9 ) | 106 ( 29.8 ) | 43 (38.7 ) | 0.0769^^ | 84 ( 40.4 ) | 43 ( 41.3 ) | 41 ( 39.4 ) | 0.7775^^ |
| **Estimated Glomerular Filtration Rate (eGFR) Baseline, Median (Q1,Q3)** | 75.0 (44.00, 97.00) | 76.0 (43.00, 97.00) | 74.0 (51.00, 96.00) | 0.9451^ | 74.0 (44.00, 94.00) | 74.0 (40.00, 92.00) | 73.0 (49.00, 96.00) | 0.4921* |
| **Acute Kidney Injury (AKI) within 24 hours of ICU-admission, n (%)** | 129 ( 27.2 ) | 101 ( 27.9 ) | 28 ( 25.0 ) | 0.5467^^ | 61 ( 29.3 ) | 33 ( 31.7 ) | 28 ( 26.9 ) | 0.4463^^ |
| **Mechanical Ventilation within 24 hours of ICU-admission, n (%)** | 337 ( 70.5 ) | 268 ( 73.8 ) | 69 ( 60.0 ) | 0.0046^^ | 126 ( 60.6 ) | 64 ( 61.5 ) | 62 ( 59.6 ) | 0.7766^^ |
| **Oxygenation Index (OI)** | 15.9 (7.42, 29.27) | 15.7 (7.42, 26.45) | 18.0 (7.98, 39.08) | 0.2795^ | 17.3 (7.98, 29.22) | 16.3 (8.70, 26.50) | 17.3 (7.98, 32.72) | 0.3044^ |
| **Inotropes/vasopressors use within 24 hours of admission)** | 97 ( 20.4 ) | 76 ( 21.0 ) | 21 ( 18.6 ) | 0.5790^^ | 37 ( 17.8 ) | 17 ( 16.3 ) | 20 ( 19.2 ) | 0.5865^^ |
| **Lactic acid Baseline (mmol/L), Median (Q1,Q3)** | 1.7 (1.30, 2.44) | 1.7 (1.29, 2.44) | 1.6 (1.35, 2.58) | 0.5655^ | 1.7 (1.30, 2.54) | 1.6 (1.14, 2.46) | 1.7 (1.37, 2.79) | 0.2998^ |
| **Platelets count Baseline (10^9/L), Median (Q1,Q3)** | 238.0 (183.00, 310.00) | 243.0 (190.00, 312.00) | 218.5 (164.50, 307.00) | 0.0639^ | 230.0 (174.00, 307.00) | 244.0 (182.00, 307.00) | 217.5 (155.00, 307.00) | 0.1025^ |
| **Total WBC Baseline (10^9/L), Median (Q1,Q3)** | 9.5 (6.70, 12.83) | 9.7 (6.64, 13.10) | 9.3 (6.84, 12.60) | 0.6236^ | 9.6 (6.70, 13.00) | 10.1 (6.64, 14.50) | 9.1 (6.81, 12.10) | 0.1502^ |
| **International normalized ratio (INR), Median (Q1,Q3)** | 1.1 (1.03, 1.20) | 1.1 (1.02, 1.19) | 1.1 (1.06, 1.22) | 0.0029^ | 1.1 (1.04, 1.20) | 1.1 (1.04, 1.19) | 1.1 (1.06, 1.21) | 0.0220^ |
| **activated partial thromboplastin time (aPTT) Baseline (Seconds), Median (Q1,Q3)** | 30.6 (27.40, 34.10) | 30.6 (27.60, 34.00) | 30.3 (26.90, 35.55) | 0.6312^ | 30.5 (27.05, 34.35) | 30.5 (27.40, 34.00) | 30.1 (27.00, 35.90) | 0.6594^ |
| **Total bilirubin (μmol/L), Median (Q1,Q3)** | 9.6 (6.70, 13.10) | 9.0 (6.50, 12.80) | 11.0 (7.20, 14.40) | 0.0108^ | 10.0 (7.00, 13.50) | 9.8 (6.80, 12.10) | 10.8 (7.00, 14.00) | 0.1298^ |
| **Albumin Baseline (gm/L), Median (Q1,Q3)** | 33.0 (29.00, 36.00) | 33.0 (29.00, 36.20) | 31.0 (28.00, 35.90) | 0.0610^ | 32.0 (28.00, 35.80) | 32.3 (28.00, 35.80) | 31.0 (28.00, 35.60) | 0.4075^ |
| **Creatine phosphokinase (CPK) baseline (U/l), Median (Q1,Q3)** | 169.0 (73.00, 404.00) | 170.5 (79.00, 427.00) | 140.5 (62.00, 370.50) | 0.1767^ | 179.0 (74.00, 409.00) | 220.0 (91.00, 477.00) | 149.0 (57.00, 378.50) | 0.1081^ |
| **C-reactive protein (CRP) baseline (mg/l), Median (Q1,Q3)** | 131.0 (55.20, 193.00) | 136.0 (73.00, 193.00) | 76.0 (11.60, 192.00) | 0.0040^ | 109.0 (23.60, 199.50) | 120.5 (37.60, 197.00) | 72.0 (11.60, 202.00) | 0.1678^ |
| **Procalcitonin (ng/ml), Median (Q1,Q3)** | 0.3 (0.14, 1.24) | 0.3 (0.13, 1.03) | 0.5 (0.17, 1.84) | 0.1761^ | 0.5 (0.16, 1.75) | 0.5 (0.14, 1.75) | 0.5 (0.16, 1.96) | 0.7747^ |
| **Fibrinogen Level baseline (gm/l), Median (Q1,Q3)** | 5.5 (3.73, 7.05) | 5.5 (3.58, 7.28) | 5.2 (3.90, 6.93) | 0.9503^ | 5.0 (3.37, 6.93) | 5.0 (3.01, 7.01) | 5.1 (3.90, 6.93) | 0.4356^ |
| **D-dimer Level baseline (mg/l), Median (Q1,Q3)** | 1.3 (0.74, 3.10) | 1.5 (0.77, 3.38) | 1.2 (0.62, 2.08) | 0.0429^ | 1.3 (0.77, 2.49) | 1.6 (0.88, 3.10) | 1.2 (0.63, 2.12) | 0.0225^ |
| **Ferritin Level baseline (ug/l), Median (Q1,Q3)** | 775.9 (358.80, 1650.00) | 792.6 (364.00, 1650.00) | 715.0 (305.80, 1650.00) | 0.4744^ | 852.6 (397.30, 1650.00) | 1051.0 (474.00, 1848.00) | 733.3 (328.60, 1650.00) | 0.2010^ |
| **Blood glucose level Baseline (mmol/L), Median (Q1,Q3)** | 11.3 (8.08, 15.90) | 11.4 (8.30, 16.30) | 11.3 (7.50, 14.10) | 0.1997^ | 11.6 (8.28, 15.70) | 12.0 (8.42, 16.70) | 11.4 (8.03, 13.90) | 0.1145^ |
| **Lowest PaO2/FiO2 ratio within 24 hours of admission, Median (Q1,Q3)** | 79.6 (59.70, 129.60) | 81.2 (58.33, 129.10) | 78.8 (63.88, 140.40) | 0.4254^ | 80.5 (60.62, 127.60) | 83.1 (60.00, 124.60) | 79.5 (63.94, 141.70) | 0.5545^ |
| **Respiratory Rate (RR) Baseline (Breath per minute), Median (Q1,Q3)** | 28.0 (22.00, 33.00) | 28.0 (23.00, 33.00) | 27.0 (22.00, 33.00) | 0.9773^ | 27.0 (23.00, 33.00) | 27.5 (24.00, 33.00) | 27.0 (22.00, 32.00) | 0.5976^ |
| **Maximum body temperature Baseline(C°), Median (Q1,Q3)** | 37.3 (37.00, 38.00) | 37.2 (37.00, 38.00) | 37.5 (37.00, 38.20) | 0.1170^ | 37.3 (37.00, 38.10) | 37.2 (37.00, 38.00) | 37.5 (37.00, 38.20) | 0.1338^ |
| **Pharmacological DVT prophylaxis use during ICU stay, n (%)** | 435 ( 90.6 ) | 329 ( 90.1 ) | 106 ( 92.2 ) | 0.5134^^ | 193 ( 92.8 ) | 98 ( 94.2 ) | 95 ( 91.3 ) | 0.4213^^ |
| **Patient received nephrotoxic drugs/material during ICU stay, n (%)** | 385 ( 80.9 ) | 298 ( 82.5 ) | 87 ( 75.7 ) | 0.1015^^ | 158 ( 76.0 ) | 79 ( 76.0 ) | 79 ( 76.0 ) | >0.9999^^ |
| **Comorbidity, n (%)** |  |  |  |  |  |  |  |  |
| Atrial fibrillation (A Fib) | 13 ( 2.7 ) | 11 ( 3.0 ) | 2 ( 1.7 ) | 0.4628** | 5 ( 2.4 ) | 3 ( 2.9 ) | 2 ( 1.9 ) | 0.6508** |
| Heart Failure (HF) | 31 ( 6.5 ) | 24 ( 6.6 ) | 7 ( 6.1 ) | 0.8526^^ | 11 ( 5.3 ) | 5 ( 4.8 ) | 6 ( 5.8 ) | 0.7567^^ |
| Hypertension (HTN) | 273 ( 56.9 ) | 205 ( 56.2 ) | 68 ( 59.1 ) | 0.5754^^ | 119 ( 57.2 ) | 57 ( 54.8 ) | 62 ( 59.6 ) | 0.4835^^ |
| Diabetes mellitus (DM) | 295 ( 61.5 ) | 225 ( 61.6 ) | 70 ( 60.9 ) | 0.8817^^ | 128 ( 61.5 ) | 63 ( 60.6 ) | 65 ( 62.5 ) | 0.7756^^ |
| Dyslipidemia (DLP) | 85 ( 17.7 ) | 68 ( 18.6 ) | 17 ( 14.8 ) | 0.3459^^ | 39 ( 18.8 ) | 22 ( 21.2 ) | 17 ( 16.3 ) | 0.3744^^ |
| Ischemic heart disease (IHD) | 38 ( 7.9 ) | 28 ( 7.7 ) | 10 ( 8.7 ) | 0.7227^^ | 21 ( 10.1 ) | 11 ( 10.6 ) | 10 ( 9.6 ) | 0.8180^^ |
| Chronic kidney disease (CKD) | 46 ( 9.6 ) | 36 ( 9.9 ) | 10 ( 8.7 ) | 0.7107^^ | 21 ( 10.1 ) | 12 ( 11.5 ) | 9 ( 8.7 ) | 0.4899^^ |
| Cancer (any type) | 11 ( 2.3 ) | 9 ( 2.5 ) | 2 ( 1.7 ) | 0.6498** | 2 ( 1.0 ) | 1 ( 1.0 ) | 1 ( 1.0 ) | >0.9999** |
| Acute Coronary Syndrome (ACS) | 7 ( 1.5 ) | 6 ( 1.6 ) | 1 ( 0.9 ) | 0.5459** | 3 ( 1.4 ) | 2 ( 1.9 ) | 1 ( 1.0 ) | 0.5609** |
| Deep vein thrombosis (DVT) | 1 ( 0.2 ) | 0 ( 0.0 ) | 1 ( 0.9 ) | 0.0745** | 1 ( 0.5 ) | 0 ( 0.0 ) | 1 ( 1.0 ) | 0.3161** |
| Pulmonary embolism (PE) | 3 ( 0.6 ) | 2 ( 0.5 ) | 1 ( 0.9 ) | 0.7027** | 1 ( 0.5 ) | 0 ( 0.0 ) | 1 ( 1.0 ) | 0.3161** |
| Liver disease (any type) | 7 ( 1.5 ) | 5 ( 1.4 ) | 2 ( 1.7 ) | 0.7733** | 4 ( 1.9 ) | 2 ( 1.9 ) | 2 ( 1.9 ) | >0.9999** |
| Stroke | 26 ( 5.4 ) | 15 ( 4.1 ) | 11 ( 9.6 ) | 0.0242^^ | 15 ( 7.2 ) | 4 ( 3.8 ) | 11 ( 10.6 ) | 0.0606^^ |
| *T Test / ^ Wilcoxon rank sum test is used to calculate the P-value.  ^^ Chi square/ ** Fisher’s Exact teat is used to calculate P-value. | | | | | | | | |
